# Specific Bacilli in Bilious Vomiting in Typhoid

**Published:** 1916-11

**Authors:** 


**Specific Bacilli in Bilious Vomiting in Typhoid.** Jean Bauer, Hautefeuille and J. Sylvestre, Soc. Med. des Hop., Jan. 26, while agreeing that blood cultures are indispensable, state that bacilli are found in the bilious vomitus of certain cases of typhoid and paratyphoid and then afford a prompt diagnosis. The bacilli may merely have been eliminated by the liver or they may be colonizations destined to produce specific angiocholecystitis.

